# Mouse models of human disease: An evolutionary perspective

**DOI:** 10.1093/emph/eow014

**Published:** 2016-05-21

**Authors:** Robert L. Perlman

**Affiliations:** Department of Pediatrics, The University of Chicago, 5841 S. Maryland Ave, MC 5058, Chicago, IL 60637, USA

**Keywords:** allometry, cancer, gene networks, life history, model organisms

## Abstract

The use of mice as model organisms to study human biology is predicated on the genetic and physiological similarities between the species. Nonetheless, mice and humans have evolved in and become adapted to different environments and so, despite their phylogenetic relatedness, they have become very different organisms. Mice often respond to experimental interventions in ways that differ strikingly from humans. Mice are invaluable for studying biological processes that have been conserved during the evolution of the rodent and primate lineages and for investigating the developmental mechanisms by which the conserved mammalian genome gives rise to a variety of different species. Mice are less reliable as models of human disease, however, because the networks linking genes to disease are likely to differ between the two species. The use of mice in biomedical research needs to take account of the evolved differences as well as the similarities between mice and humans.

If you have cancer and you are a mouse, we can take good care of you. Judah Folkman [1]

## INTRODUCTION

Because of their phylogenetic relatedness and physiological similarity to humans, the ease of maintaining and breeding them in the laboratory, and the availability of many inbred strains, house mice, *Mus musculus*, have long served as models of human biology and disease [2]. Genomic studies have highlighted the striking genetic homologies between the two species [3, 4]. These studies, together with the development of methods for the creation of transgenic, knockout, and knockin mice, have provided added impetus and powerful tools for mouse research, and have led to a dramatic increase in the use of mice as model organisms. Studies on mice have contributed immeasurably to our understanding of human biology [5]. All too often, however, mice respond to experimental interventions in ways that differ markedly from humans. Endostatin, the anticancer drug alluded to in the epigraph, is but one of many treatments that cure cancer in mice but have limited effectiveness in humans [6]. Indeed, the majority of oncology drugs that enter clinical trials never reach the marketplace. There are many reasons for the high failure rate of drug development, but the limitations of the animal models used in drug testing are an important factor [7]. Many substances that are carcinogens in mice are not carcinogenic in humans—and vice versa [8]. Moreover, mouse strains that were created to mimic human genetic diseases frequently have phenotypes that differ from their human counterparts [9]. Because of the assumption that mice will serve as reliable models for humans, differences between the two species are often greeted with surprise as well as dismay. But these differences should not elicit surprise; indeed, they should be expected. The lineages leading to modern rodents and primates are thought to have diverged from a common ancestral species that lived some 85 million years ago [10]. Since that time, species in these lineages evolved in and became adapted to very different environments. Our evolved developmental processes produce different kinds of organisms from similar component parts. Differences between mice and humans may be due to selection or drift, acting over the eons of evolutionary time or more recently during the creation and breeding of laboratory mouse strains [11].

## SIZE

The most obvious and perhaps the most fundamental difference between mice and humans is size: humans are roughly 2500 times larger than mice. Size influences many aspects of an organism’s interactions with its environment, including its ability to acquire food, to avoid predators and to attract mating partners, and so has important effects on fitness; in the words of J. B. S. Haldane, organisms must be “the right size” [12]. As the lineages leading to mice and humans evolved, there was presumably selection for organisms that were the right size for their environments. Given its importance, size itself was probably a major target of natural selection [13, 14]. But a host of traits are correlated with size, and during the course of rodent and primate evolution, these traits evolved together with size. Two prominent sets of traits that are correlated with size are metabolic rate and life history strategy [15].

## METABOLIC RATE

Metabolic rates of placental mammals are closely correlated with size. The relationship between basal metabolic rate (in kcal/day) and body mass (in kg) is usually taken as BMR = 70 × Mass^0.75^ [16]. Thus, a 30-g mouse has a specific metabolic rate (metabolic rate per gram of tissue) roughly seven times that of a 70-kg human [15]. There is continuing controversy about the reasons for the relationship between size and metabolic rate, and about the value of the allometric exponent [17]. The increased specific metabolic rate of small mammals is presumably related, at least in part, to size-dependent differences in heat loss and in requirements for thermoregulation, and is characterized by increases both in nutrient supply (capillary density) [18] and in nutrient demand (mitochondrial density) [19] in tissues of small animals; since nutrient supply and demand have coevolved and develop together during ontogeny, they are closely matched [20]. Differences in metabolic rate between mice and humans are correlated with many anatomic, physiologic and biochemical differences. Mice have relatively higher amounts of metabolically active tissues, such as liver and kidney, and relatively less inactive tissue, such as bone; in addition, mice have larger deposits of brown fat, which plays a critical role in heat production and thermoregulation. Mouse cells differ from human cells not only in mitochondrial density and metabolic rate, but also in the fatty acid composition of their membrane phospholipids; specifically, membranes in mouse cells have a higher content of the polyunsaturated (and readily oxidizable) fatty acid docosahexaenoic acid [21]. Mice have higher rates of production of reactive oxygen species and suffer higher rates of oxidative damage than do humans. All of these differences presumably evolved in association with selection for differences in size or in association with some other trait that is correlated with body mass, such as life history and rate of aging.

## LIFE HISTORY

Size is also associated with a suite of life history traits, including age at reproductive maturity, length of gestation, litter size, birth interval, fraction of energy devoted to reproduction, and, perhaps most importantly, life expectancy. Female wild mice reach sexual maturity in a matter of 6–8 weeks, have a gestation length of 19–20 days and a litter size of 5–8, and produce multiple litters a year. Many laboratory mouse strains have been selected for increased fertility; they reach sexual maturity earlier and produce larger litters than do wild mice [22]. Mice, like other rodents, invest a much larger proportion of their energy in reproduction than do humans [23]. Both wild and laboratory mice have life spans of about 3–4 years, but wild mice have a much shorter life expectancy (less than a year, depending of course on environmental conditions) than do laboratory strains, which typically live several years [22]. Again, the differences in life history strategies between humans and mice are correlated with, and are probably related to, differences in size.

## DIETS, MICROBIOMES AND PATHOGENS

Evolved differences in murine and human diets are also associated with pervasive differences in the biology of the two species. Although both mice and humans are omnivores, wild mice seem preferentially to consume unprocessed grains and cereals. Mice have large and continuously growing incisors that enable them to eat these foods. Presumably because their ancestors’ diets were low in ascorbic acid, mice have retained the ability to synthesize this essential cofactor; humans, in contrast, have lost this ability and so we now require exogenous vitamin C. And presumably because of their ancestors’ ingestion of different xenobiotics, mice and humans have different complements of cytochrome P450 enzymes and different patterns of xenobiotic metabolism [24, 25]. At least in part for this reason, toxicology testing in mice has been a poor predictor of human toxicity [26]. More importantly, mice have different microbiomes [27] and have coevolved with different sets of pathogens than have humans. The anatomy of the gastrointestinal track differs between the two species [27]. The ratio of length of the small intestine to that of the colon is greater in mice than in humans, mice have a prominent cecum, and they lack an appendix. In mice, the cecum is an important site for the microbial fermentation of undigested foods. Thus, the two species provide different environments that apparently support the growth of different gastrointestinal microbiota. Moreover, mice have significant amounts of bronchus-associated lymphoid tissue, which has been interpreted to indicate that, because they live close to the ground, they face increased exposure to respiratory pathogens in droplets or particles from the soil [28]. The differences between mice and humans are not only genetic and epigenetic, but also reflect features of their environments, especially their ecological interactions with other species (food sources, microbiota, pathogens, etc.) that are reliably transmitted from generation to generation and affect the course of development.

## DIFFERENCES DUE TO THE DOMESTICATION AND BREEDING OF HOUSE MICE

During the course of murine and human evolution, our ancestors underwent selection for—and so mice and humans now differ in—many other traits, including circadian rhythm (wild mice are nocturnal), sensory systems (mice rely heavily on olfaction, hearing and touch), cognitive development, reproductive behavior and patterns of social organization. Moreover, the domestication and breeding of the laboratory mouse strains that are commonly used in biomedical research have increased the differences between the biology of these strains and that of wild mice, let alone human biology. Many laboratory mouse strains were derived from fancy mice, which had been kept as pets for centuries. These strains were derived largely from the subspecies *M. musculus domesticus*, which, for unknown reasons, has an exceptionally high rate of robertsonian chromosomal translocations [29]. Initially, domestication entailed selection for such traits as docility and the ability to thrive and reproduce in confinement. Later, as mouse breeding became a commercial enterprise, breeders selected for traits associated with increased reproduction, including early sexual maturity and the production of frequent and large litters [30].

A major impetus for the development of inbred mouse strains was to study the genetic basis of cancer; strains were created that differed in their susceptibility to transplanted tumors or in the incidence of spontaneous neoplasms [31]. These inbred strains have yielded many important insights into cancer biology. Nonetheless, cancer and other diseases in laboratory mice that were selected because they develop (or are resistant to) these diseases may differ from the cognate diseases found in wild mice, as well as from diseases in humans. Common strains of laboratory mice have come to differ from wild mice in a host of traits. Some of these differences, such as increased fertility, can be understood as the result of selection, while the reasons for other differences are not clear [30]. Finally, the genetic homogeneity that makes these strains valuable in the laboratory means, of course, that they lack the genetic variation that characterizes outbred wild populations.

Given the many differences in the biology of mice and humans, it is not surprising that the patterns of disease differ in the two species. The causes of death of feral house mice depend on the environment. Many are killed by predators, and in harsh environments starvation and hypothermia are major causes of death [22]. In the laboratory, mice live longer; there, cancer is a major cause of death, while cardiovascular disease is negligible. The distribution of tumors differs between mice and humans; most murine tumors are of mesenchymal origin, while human tumors arise mainly from epithelial cells. There are many other differences between mouse and human cancers, and many differences between mouse and human cells that appear to contribute to these differences [8, 32–34]. For example, laboratory mouse strains have much longer telomeres than do humans and express telomerase in their somatic cells throughout life. This difference may help to explain why, *in vitro***,** mouse cells undergo spontaneous transformation at much higher rates than do human cells.

Some of the differences between mice and humans are relatively easy to rationalize. As discussed below, differences in the function of the immune system have almost certainly evolved in response to differences in pathogen exposure and in life expectancy [28]. Other differences, such as differences in genomic imprinting, are harder to understand [35]. Additional phylogenetic analyses and functional genomic studies will be necessary to determine which of the differences between mouse and human biology are related to differences in size, either because they are associated with metabolic rate or with life history strategy, which are due to other changes that accompanied the evolutionary divergence of these species, and which have resulted from the selective breeding of laboratory mice.

## IMPLICATIONS OF SPECIES DIFFERENCES FOR MOUSE RESEARCH

The use of model organisms in biological research is based on the concept of unity in biology, a concept expressed most famously in Jacques Monod and François Jacob’s aphorism, “Anything found to be true of *E. coli* must also be true of elephants” [36]. But biology is characterized by diversity as well as unity; evolution is “descent with modification” [37]. The art of choosing model organisms involves recognizing the properties of these organisms that they are likely to share with organisms of other species—especially, for biomedical research, humans [38]. Monod and Jacob were concerned with genetic regulatory mechanisms and other basic biological processes that must have arisen very early in the evolutionary history of living organisms and so are similar in bacteria and in mammals. Mice have served and will continue to serve as valuable models for the study of basic biological processes that, in Wimsatt's terms, became developmentally entrenched before the rodent and primate lineages diverged and have been conserved during the separate evolutionary histories of mice and humans [39].

Studies of the immune system highlight both the value of mouse research in elucidating common features of mammalian biology as well as the limitations of translating this research in areas in which humans are likely to differ from mice. Research on mice has contributed greatly to our knowledge of the adaptive immune system; mouse research has led to the discovery of the major histocompatibility complex genes and the T cell receptor, and to our understanding of the regulation of antibody synthesis and many other features of the immune system [40]. But there are many differences between the mouse and human immune systems, such that much research on immunological diseases in mice is not transferable to humans, and many immunologists are now calling for a return to the study of human immunology [28, 40–42]. From an evolutionary perspective, this is understandable. The adaptive immune system evolved in jawed fish some hundreds of million years before the evolution of mammals. Many features of this ancestral immune system, including antigen recognition, generation of antibody diversity, clonal selection, and immunological tolerance, are critical for survival and have been maintained in most or all of the descendants of these early vertebrates. On the other hand, species differences in the mechanisms for the maintenance of memory T cells must have evolved in response to the evolution of different life spans. Moreover, specific features of the immune system evolve rapidly, as host species coevolve with their pathogens and commensal microbiota [41]. Since humans and mice harbor different sets of pathogens and microbiomes, it is not surprising that host–pathogen and host–microbiome coevolution has led to differences between the human and mouse immune systems.

The fact that the highly conserved mammalian genome can give rise to a wide variety of different species indicates that the relationships between genotype and phenotype differ among mammalian species. Comparisons between mice and humans are invaluable for understanding the developmental mechanisms that lead to such different genotype–phenotype relationships. Some of the genetic differences between mice and humans are differences in coding sequences, which give rise to proteins with different properties. For example, mouse hemoglobin has a lower affinity for O_2_ than does human hemoglobin, which facilitates the dissociation of O_2_ from hemoglobin in peripheral tissues and helps to support the higher metabolic rate in mice. Perhaps more importantly, however, are differences in the genetic or epigenetic regulation of gene expression in these species. The expression of potassium channel genes in the heart exemplifies these differences. Mice have a heart rate of ∼600 beats/min, while humans have a resting heart rate of ∼70 beats/min. This difference in heart rate entails that the cardiac action potential be much shorter in mice than in humans. Indeed, the repolarization phase of the cardiac action potential, which is due to outward K^+ ^currents, is much shorter in mice [43]. This difference is due to different contributions of various K^+ ^currents, which in turn are presumably due to differences in expression of K^+ ^channel genes in the two species. Evolved differences in the regulation of gene expression are important because they may involve the rewiring of gene (or protein) networks. Gene networks in mice and humans have similar numbers of nodes (genes) but the connectivity of the nodes in these networks, and the relationships between genes and phenotypes, differ between the two species [44–46]. The different network architectures and different genotype–phenotype relationships between mice and humans mean that the relationships between genotype and disease are also likely to differ in these two species. Perturbations of gene and protein networks by environmental manipulation as well as by mutation are likely to have different effects on diseases as well as on other phenotypes in mice than in humans. In short, mice are problematic models for understanding human disease.

There are other good reasons to pursue research on mice. Although house mice are not a major source of human disease, they can transmit lymphocytic choriomeningitis virus and perhaps other pathogens to humans, and other rodent species are important reservoirs for zoonoses. Research on mice may yield information that will help to prevent or ameliorate these diseases. Finally, mice should be studied for their own sake, to understand their biology and to maintain the health of pet mice, laboratory mice, and wild mice.

Unfortunately, despite the many attempts to translate the results of mouse research to humans, we still cannot specify in advance which research in mice is likely to benefit or shed light on human biology and health. For the most part, we have only anecdotal information about studies in mice that translated to humans and those that did not. We need more systematic collection, reporting and analysis of mouse research (and research on other “model organisms”) to figure out what works and what does not. Until we have that information, we need to be more critical in pursuing mouse research and in making claims about the applicability of this research to humans.

In addition to problems resulting from the evolved differences between mice and humans, other aspects of mouse research have compromised the value of this research and have further complicated the extrapolation of mouse research to humans. Thus, e.g., laboratory mice are often housed at temperatures below their thermoneutral zone, and as a result are cold-stressed, sleep deprived, and hypertensive [47]. The biology of laboratory mice may also be affected by their housing in same-sex groups and their lack of opportunities for physical exercise. Although mice are often used as models of diseases of aging, for logistical and financial reasons most mouse research is carried out on young animals. And although mouse cells are more sensitive to oxygen damage than are human cells, cell culture studies are often carried out in 20% oxygen, which is non-physiological and is more damaging to mouse cells than to human cells [48]. Finally, there are no agreed upon standards for the design, analysis, or publication of mouse research (or research with other model organisms). The statistical analysis of studies of mice and other animals is often substandard, and there may be important publication biases because negative results may not get published [49, 50]. All of these problems need to be addressed before studies on mice can be properly interpreted and extrapolated to humans.

## FINAL COMMENTS

Despite all of the documented differences between mice and humans, and despite the history of “errors in translation” in the application of research on mice to humans, reports of research on mice are frequently accompanied by unwarranted and misleading claims, such as “Furthering our understanding of mouse X should provide novel insights into human Y.” Such claims raise false hopes and are ultimately self-defeating, in that they waste resources and increase public skepticism concerning the value of biomedical research. Indeed, the problems of translating research on mice and other model organisms to humans have led a number of scientists to question the value of this research [51–53]. Furthermore, critical discussions of animal experimentation are routinely distorted by “animal rights” activists to support their belief that this experimentation should be stopped. These intrusions, however unwelcome, should not stifle discussion. For reasons mentioned above, research on mice (and other species) is essential and should be supported. This research should, however, be designed and interpreted with appropriate appreciation of the evolved differences as well as the similarities between *M. musculus* and *H. sapiens*.
